# Healthcare-associated infections in the context of the pandemic

**DOI:** 10.3389/frhs.2023.1288033

**Published:** 2023-11-28

**Authors:** Mohammed S. Razzaque

**Affiliations:** Department of Pathology, Lake Erie College of Osteopathic Medicine, Erie, PA, United States

**Keywords:** COVID-19, healthcare-associated infections, infection prevention, patient safety, outcomes

Healthcare-associated infections (HCAIs) occur in individuals while receiving medical care in a healthcare facility. These infections are often preventable. According to the U.S. Center for Disease Control and Prevention (CDC), around 1.7 million hospitalized patients acquire HCAIs per year while being treated for other health-related problems; one out of these seventeen infected patients die as a result of HCAIs (1), which is one of the top ten causes of death in the U.S. Around 7% of patients in high-income nations and 10% in emerging and developing nations acquire HCAIs, and 10% of those patients pass away (1). The rate of HCAIs is higher among intensive care unit (ICU) patients, mostly due to their immunocompromised status (2, 3). Of relevance, the higher risk of mortality among patients in the ICU is not only limited to their primary illness but is often amalgamated with HCAIs. The COVID-19 pandemic has highlighted the danger of HCAIs and the need for rigorous infection control measures in healthcare settings. A survey of 11,282 patients in various U.S. hospitals identified *Clostridium difficile* as the major cause of HCAIs (4). Another study on a large cohort found more than 2 million new patients developing HCAIs with antimicrobial resistance to *Klebsiella pneumoniae* and *Acinetobacter* species per year, in the European Union and European Economic Area (5).

**Figure 1:**
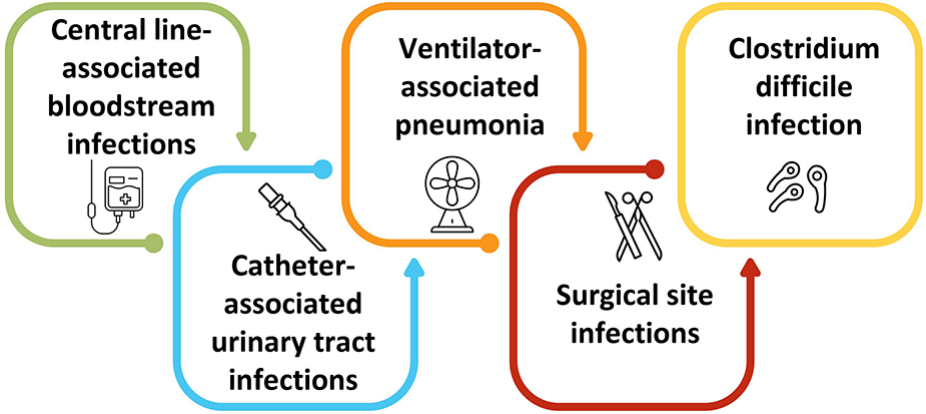
Partial list of a few common causes of HCAIs.

Bloodstream infection, urinary tract infection, surgical site infection, and pneumonia are identified as the most common causes of HCAIs (Figure 1) (6, 7). Bloodstream infections in ICU and hemodialysis centers are common, and around US$ 1.8 billion was spent in a decade in early 2000 to save more than 25,000 patients (8). Surgical site infection is a common postoperative complication with higher morbidity and mortality that comes with a financial burden to the patients and the care providers (9). Urinary tract infections, particularly catheter-induced infections, are among the most common causes of HCAIs, comprising around 40% of HCAIs, with higher fatality (10). Implants and prostheses can also induce HCAIs. The increased rate of HCAIs with higher numbers of antimicrobial resistance significantly burdens healthcare costs, particularly affecting low-resource countries more (11). Additionally, exacerbating antimicrobial resistance is another casualty of the COVID-19 pandemic (12–14). Although antibiotics are ineffective against viruses, including COVID-19, many COVID-19 patients have received antibiotics as a cautionary measure, causing an unnecessary use of antibiotics and the development of antimicrobial resistance, thereby making HCAIs more challenging to treat (15).

The available evidence suggests an association between COVID-19 and an increase in HCAIs. A primary concern during the COVID-19 pandemic is that patients were at a higher risk of acquiring the infection while receiving care in a healthcare facility. The proximity of infected healthcare individuals, the COVID-19 patients in healthcare settings, and the potential for healthcare worker-mediated transmission can increase HCAIs. A CDC analysis found a continued increase in HCAIs in U.S. hospitals during the pandemic in 2021; ventilator-associated events (VAEs) significantly increased across all types of infections (16). In a separate cross-sectional analysis of more than 5 million hospitalized patients between 2020 and 2022, the occurrence of catheter-associated urinary tract infection, central line-associated bloodstream infection, and methicillin-resistant *Staphylococcus aureus* bacteremia were found to be higher among the COVID-19 patients (17). The impact of HCAIs may vary depending on hospital practices and hospitalization period during the pandemic.

COVID-19 has also been shown to spread via bioaerosols (18). Bioaerosols are airborne particles that contain living organisms such as bacteria, viruses, and fungi (19). COVID-19 can be transmitted through bioaerosols generated when an infected person talks, coughs, or sneezes (20). Bioaerosols can be a limiting factor in reducing HCAIs, as they can spread infectious agents in various healthcare settings. Therefore, controlling bioaerosols can be an essential measure in limiting HCAIs. Proper ventilation, air filtration, hand hygiene, and toilet hygiene are some of the steps that can help reduce the concentration of bioaerosols and minimize the spread of infectious agents in healthcare settings. Proper ventilation can help decrease the concentration of bioaerosols in the air, while air filtration systems can remove bioaerosols from the air to lower the risk of microorganism transmission, including COVID-19 (21). Similarly, proper hand hygiene can help prevent the spread of infectious agents that may be present in bioaerosols, and adequate toilet hygiene and cleaning can help decrease the risk of transmission. A study found that flushing toilets, seeded with bacteria, can increase the bioaerosol concentration of a washroom to increase the spread of microorganisms (22). In the COVID-19 pandemic era, data-driven approaches to identifying the areas for improvement and implementing evidence-based practices to minimize the risk of developing HCAIs would better serve to protect patients. The COVID-19 pandemic highlighted the need for continuing education and training of infection prevention and control for healthcare workers to limits the spread of disease.

The CDC has provided guidelines for reducing HCAIs, covering primary infection prevention and control, and instructions for healthcare providers in specific settings to protect and provide safe care. The WHO advocates that all healthcare providers must wash their hands before dealing with patients. Effective hand hygiene is the most important practice to control HCAIs, which prevent the formation of colonies with multi-drug resistant pathogens (23). Poor hand hygiene compliance has shown to be one of the leading contributory factors to HCAIs, and it is estimated that improper hand hygiene by healthcare providers is responsible for about 40% of HCAIs in certain African countries (24). The WHO projected that maintenance of hand hygiene can reduce up to 50% of preventable illnesses acquired during healthcare delivery; a significant decrease in the rate of HCAIs was noted when hand hygiene compliance improved (25). As mentioned, a simple measure like handwashing is considered to be the single most effective action to stop the spread of infection, and such a measure becomes increasingly critical in the context of the COVID-19 pandemic. Furthermore, poor cleaning of the hospital surfaces is linked to HCAIs such as the transmission of the potentially fatal methicillin-resistant *Staphylococcus aureus* (26). Ongoing surveillance, education, and training of healthcare workers remain vital in reducing the incidence of HCAIs during the COVID-19 pandemic (27).

As stated, HCAIs constitute a significant health concern for both healthcare providers and recipients. During the COVID-19 pandemic, the rate of HCAIs is alarming and associated with prolonged hospitalizations, increasing morbidity and mortality (28). With evolving microorganisms and emerging microbial drug resistance, a dynamic change in healthcare practice would require ensuring hospital safety, reducing the occurrence of HCAIs, and minimizing the financial burden on individuals and society. Despite yearly spending between US$ 28 and US$ 45 billion for controlling HCAIs, around 90,000 patients die in the U.S. related to HCAIs (29, 30). The National Healthcare Safety Network found that COVID-19 patients are more vulnerable to HCAIs and require additional protective measures (17). During the COVID-19 pandemic, healthcare facilities must implement effective infection control policies and education initiatives to lower the risk of HCAIs and protect patients from harm (Box 1). Moreover, healthcare workers need to undergo training on infection prevention and control measures to minimize the risk of transmission to ensure patient safety and improve health outcomes. Healthcare facilities can provide safe and compassionate patient care (31), even amid the COVID-19 pandemic, by focusing on infection prevention and control.

**Box 1 Specific preventive measures to reduce HCAIs.**

• Proper hand hygiene
• Adequate cleaning and disinfection of equipment and facilities
• Appropriate use of antibiotics
• Use of catheters and other medical devices selectively
• Vaccination of healthcare workers and patients
• Control of bioaerosol spread
